# Reply to: “Impact of marine processes on flow dynamics of northern Antarctic Peninsula outlet glaciers” by Rott et al.

**DOI:** 10.1038/s41467-020-16685-9

**Published:** 2020-06-11

**Authors:** Peter A. Tuckett, Jeremy C. Ely, Andrew J. Sole, Stephen J. Livingstone, Benjamin J. Davison, J. Melchior van Wessem

**Affiliations:** 1 Department of Geography, The University of Sheffield, Sheffield, UK; 2 School of Geography and Sustainable Development, University of St Andrews, St Andrews, UK; 3 Institute for Marine and Atmospheric Research Utrecht, Utrecht University, Utrecht, The Netherlands

**Keywords:** Climate change, Cryospheric science

**Replying to** Rott et al. *Nature Communications* 10.1038/s41467-020-16658-y (2020)

In Tuckett et al.^1^, we report short-term speed-up events of Antarctic Peninsula outlet glaciers. Modelled surface melting, observations of surface meltwater, and speed-up event characteristics led us to propose speed-ups were a consequence of meltwater reaching the ice-bed interface; a meltwater hypothesis. Rott et al.^2^ replicate the velocity data and show that during one event, sea-ice conditions change ~90 km from three glaciers, and at the front of another, leading Rott et al.^2^ to propose a sea ice hypothesis: that sea-ice movement away from glacier fronts reduces back-stress triggering acceleration. Simultaneously Rott et al.^2^, argue that the ice velocity observations are biased due to measurement artefacts. Here, we defend the meltwater hypothesis, present evidence against the sea-ice hypothesis, and examine potential bias in our glacier velocity measurements.

Although sea-ice evacuation is coincident with the March 2018 speed-up event observed in Tuckett et al.^1^, changes in sea-ice characteristics are not synchronous across all studied glaciers. Movement of sea ice, driven by Foehn winds (Fig. 1a), at the front of Drygalski Glacier, and distal to Hektoria, Cayley and Jorum glaciers (past ~90 km of unchanged multi-annual fast pack ice), during the March 2018 event was presented by Rott et al.^2^ as evidence for the sea-ice hypothesis. Foehn winds also induce surface melting. We interpret this melting as the trigger of the speed-up event, and the sea-ice movement as a by-product of Foehn winds for the following reasons. First, sea-ice cover increases near Cayley glacier during this event (Supplementary Table 1) while during other events, wind speeds are lower (Fig. 1a) and glacier-adjacent sea-ice conditions remain unchanged (Fig. 1b). Second, all three speed-up events have a corresponding spike in surface melting (Fig. 1a). Third, we find it unlikely that changes in sea ice, far from the glacier front (~90 km), can trigger a speed-up event. Finally, the sea-ice hypothesis cannot explain how glaciers return to pre-event velocities^1^, as the sea ice does not rapidly reform to its pre-breakup structure (Fig. 1c). Conversely, the meltwater hypothesis incorporates an explanation for the return to pre-event ice velocity: namely that the subglacial drainage system adapts to accommodate the extra water flux, thereby reducing basal water pressure^3^.

**Fig. 1:**
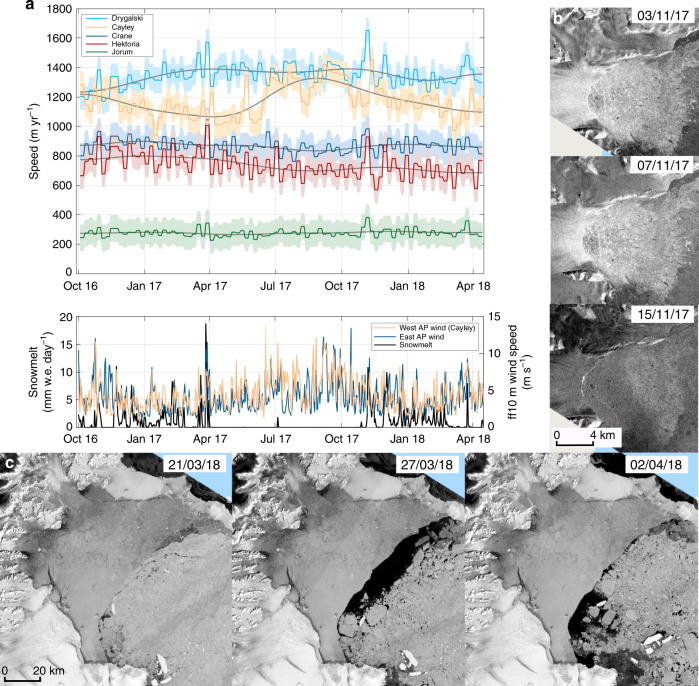
Sea-ice conditions during speed-up events. **a** Time series of glacier velocity, modelled melt and 10 m wind speed. Shaded areas are estimates of error (91 m/yr; Supplementary Note 4). **b** Sea-ice conditions at Drygalski glacier during November 2017 remain visibly unchanged throughout this event. **c** Sea ice of the Larsen B Embayment, March 2018. Note how the sea ice at the end of the speed-up (02/04/2018) does not return to the configuration prior to the speed-up (21/03/2018). We never observe a return to pre-event sea-ice structure and extent (Supplementary Table 1).

Whether water is able to penetrate the Antarctic Peninsula glaciers is also questioned by Rott et al.^2^. Theory^4^, supported by extensive observations^5,6^, demonstrates that lakes 0.25–0.80 km in diameter (comparable to those observed in Tuckett et al.^1^) provide enough water to drive fracture propagation to the ice sheet bed (hydrofracture) through >1 km of cold ice. Although we have no direct observations of this process, and demonstrate that refreezing of lakes is common, hydrofracture to the bed offers the simplest explanation for the sudden disappearance of surface lakes in crevassed regions (Fig. 5 in Tuckett et al.^1^). Furthermore, speed-up events occur during large melt events (e.g. March 2017) or those which follow a prolonged period of little or no melt (e.g. November 2017 and March 2018), consistent with the theory that melt supply variability is more important than magnitude for triggering speed-ups^3^. Thus, the notion that there is insufficient water to penetrate to the bed and cause a speed-up ignores a large body of theoretical and empirical research.

Though Rott et al.^2^ are correct to raise our use of an outdated grounding line, our own up to date analysis of the grounding-line position for these glaciers shows that the majority of velocity data were obtained from grounded or partially grounded ice (Fig. 2; Supplementary Fig. 2). Excluding Hektoria (where the grounding-line position is highly uncertain due to an ice plain), 69% of our observations came from grounded ice, and 15% were located at the grounding line. For Hektoria, the majority of observations came from the ice-plain region, where ice-surface features indicate at least partially grounded ice (Fig. 2). Regardless, the location of velocity observations is irrelevant for the meltwater hypothesis. Key is that meltwater drainage through ice occurs above the grounding line, as is inferred^1^. Thus, the assertion of Rott et al.^2^ that our interpretation depends upon the assumption that velocity data come from grounded ice is false.

**Fig. 2:**
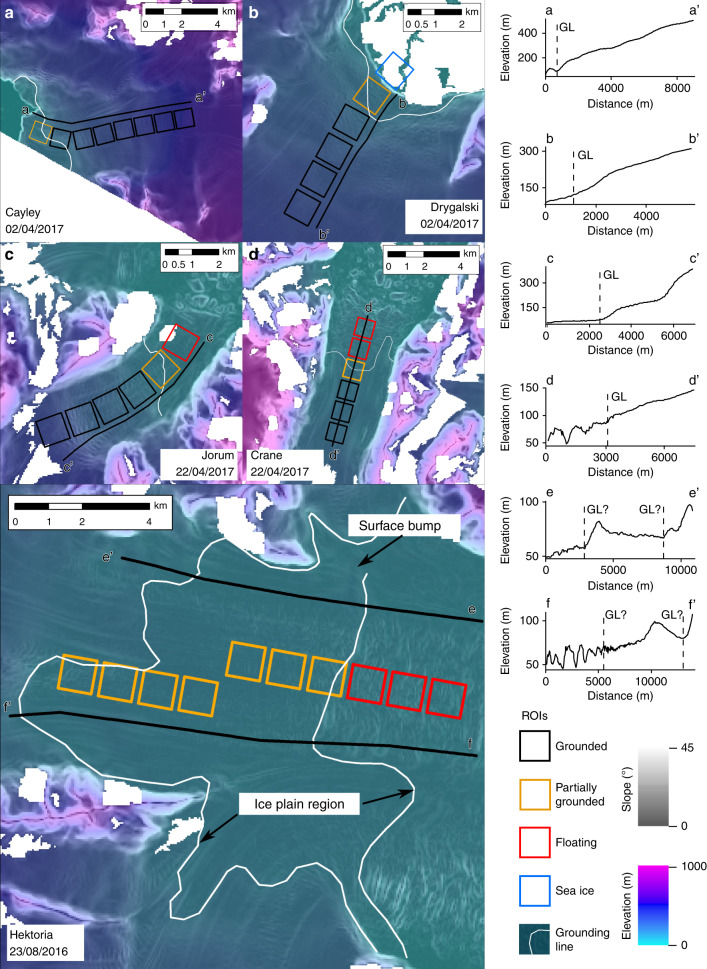
Location of the grounding line derived from REMA elevation models^10^. **a** Cayley, **b** Drygalski, **c** Jorum, **d** Crane, and **e** Hektoria glaciers. Note for Hektoria (**e**), two grounding lines are drawn, depicting an area between two breaks in slope we interpret as an ice plain. Right panel—transects along each glacier with interpreted grounding-line positions marked by GL.

We acknowledge that a spatially constant acceleration in ice flow would result in a relatively smaller change toward the glacier termini, where baseline velocities are higher. However, relative velocities do show that the relative magnitude of speed-ups decreases away from the location of water-filled crevasses and potential lake drainage, even up-flow where the baseline velocity is lower. Figure 4 in Tuckett et al.^1^ shows the relative size of the ice flow perturbation is largest near a field of crevasses observed to fill with meltwater, and the absolute magnitude of the November 2017 speed-up is greater at 9 km inland than at 4 km at Hektoria Glacier (Supplementary Fig. 4 in Tuckett et al.^1^), inconsistent with a marine driver.

The estimates of ice motion in Tuckett et al.^1^ did not account for temporal variations in radar penetration depth as the surface evolves between snow, firn, water and ice. To our knowledge, the effect this has on satellite radar velocity measurements has never been fully quantified. This introduces a bias^2^, which for our data^1^ means that a change from frozen to melted snow would induce an apparent slow-down for westward flowing glaciers and an apparent speed-up for eastward flowing glaciers (vice-versa for refreezing). Several lines of evidence suggest that this bias is smaller than the signal of speed-up events (see Supplementary Note 3). First, of our study glaciers, the bias effect should be smallest at Hektoria glacier because the ice flow direction is closest to the satellite heading angle, yet the largest speed-ups are observed here^1^. Furthermore, speed-ups similar to those identified in Tuckett et al.^1^ occur at Edgeworth Glacier, which flows roughly parallel to the satellite heading angle and so the bias should be close to zero^2^ (Supplementary Fig. 2). Moreover, speed-ups at individual glaciers vary depending on the magnitude of melt and antecedent conditions in-line with hydrological theory. Finally, the majority of speed-up events produce a net positive effect on ice velocity, whereas a net zero effect would be expected if solely due to bias. Further sources of error raised by Rott et al.^2^ are also unlikely to have a larger signal than that of the meltwater-induced speed-up events identified in ref. ^1^ (Supplementary Note 4).

Overall, we show that sea-ice and/or measurement bias are unlikely to be the main cause of the short-lived (<6 day) speed-up events observed in Tuckett et al.^1^. We note that sea-ice thinning and disintegration can cause ice flow speed-ups that are greatest near tidewater glacier and ice shelf termini and are not followed by slow-down (e.g. refs. ^7–9^), making them distinct from the events observed in Tuckett et al.^1^. The spatial and temporal pattern of ice flow variations and their relationships to environmental forcings can therefore allow the underlying chain of events to be inferred, but this is only possible with high-resolution datasets. In addition, ground-based observations would aid in elucidating and enumerating the underlying causes of the speed-up events reported in Tuckett et al.^1^.

## Supplementary information

Supplementary Information

## Data Availability

Data are available from https://figshare.shef.ac.uk/s/896c34d71a41caf5d03b. Elevation data (Fig. 2) are available from https://www.pgc.umn.edu/data/rema/.

## Code availability

Image processing to derive velocity estimates was performed using GMSTAR (https://topex.ucsd.edu/gmtsar/) and the following Matlab © functions: Particle Image Velocimetry framework: https://uk.mathworks.com/matlabcentral/fileexchange/45028-pivsuite; normalised cross-correlation with same-sized images: https://uk.mathworks.com/matlabcentral/fileexchange/29005-generalized-normalized-cross-correlation; subpixel cross-correlation peak determination: https://uk.mathworks.com/matlabcentral/fileexchange/18401-efficient-subpixel-image-registration-by-cross-correlation; image segmentation filtering: https://uk.mathworks.com/matlabcentral/fileexchange/19084-region-growing; visible structured noise filter: https://www.math.univ-toulouse.fr/~weiss/Codes/VSNR/VNSR_VariationalStationaryNoiseRemover.html.
