# Supplementary material for: Reply to: “Impact of marine processes on flow dynamics of northern Antarctic Peninsula outlet glaciers” by Rott et al
Source: Nat Commun. 2020 Jun 11;11:2970. doi: 10.1038/s41467-020-16685-9 (PMC7289856; doi:10.1038/s41467-020-16685-9)
Supplement: Supplementary file 1 — Supplementary Information [file 41467_2020_16685_MOESM1_ESM.pdf]

Supplementary information for:

Response to "Impact of marine processes on flow dynamics of northern Antarctic Peninsula outlet glaciers" by Rott et al.

by Tuckett et al.

Supplementary Notes

Supplementary Note 1: *Observations of sea ice form and extent*

Prior to, during and after each of the speed-up events, which occurred synchronously across the studied glaciers reported in [1] (March 2017, November 2017, March 2018), we visually analysed the structure of the sea ice in six locations (at the front of each glacier and at the seaward edge of the multi-year sea ice in the Larsen B embayment highlighted by [2]) using Sentinel 1 backscatter imagery. Multi-annual sea ice, new sea ice/mélange and open ocean had distinct surface textures and intensities [3]. We recorded the form and occurrence of sea ice at each of these localities (Supplementary Table 1).

Given the transient nature of the observed ice flow speed-ups, to support the sea ice hypothesis, it would have to break-up coincident with a speed-up, and then reform following a speed-up. This occurred for one locality on one occasion, when a polynya formed at the front of Drygalski glacier in March 2018, which was subsequently partially refilled by sea ice. In many cases, sea ice remained remarkably unchanged at the ice front or open water conditions persisted throughout the speed-up event. In one instance, at Cayley glacier during the March 2017 event, mélange extent increased. Overall, we detected no clear pattern in sea ice extent or structure coincident with the speed-up events (Supplementary Table 1).

Supplementary Note 2: *Determination of grounding-line position*

Rott et al. [2] use unpublished and unavailable data to claim that the majority of regions of interest (ROIs), within which velocity data were analysed by [1], are located on floating ice. To evaluate this claim, we performed additional mapping of the grounding line position, using the Reference Elevation Model of Antarctica elevation model [4]. These data were broadly coincident with our velocity data. To identify grounding line position, we used a break of slope technique (similar to that undertaken by [2]), identified visually on digital elevation models and slope maps, aided by along-flow elevation profiles.

For Jorum, Crane and Cayley glaciers, the grounding line is easily distinguishable due to the abrupt change in slope and surface texture (Fig. 2 in the main text). In the case of Hektoria glacier, highlighted in [2], the position of the grounding line is uncertain due to the presence of an ice plain. This is especially the case when using the break of slope technique, where multiple breaks in slope are apparent across a wider grounding zone rather than a distinct grounding line (Friedl et al., 2019). Figure 2 in the main text shows the grounding line position determined here in relation to the ROIs of [1]. Using our grounding line positions, 18 ROIs (50%) are

positioned on fully grounded ice, 11 (30.6%) are partially grounded (either on the grounding line or within a grounding zone), 6 ROIs (16.7%) are positioned on floating ice and 1 (2.8%) was mistakenly placed over melange/new sea ice (Fig. 2 in the main text). In the main article, we refer to figures that exclude Hektoria. It is clear that the grounding-line position of Hektoria Glacier is highly uncertain due to the presence of an ice-plain (Fig. 2 in the main text; [5]).

Supplementary Note 3 **- Ice velocity and dB changes during surface melt events**

As pointed out by [2], changes in surface moisture affect radar penetration, altering the phase centre depth. This introduces a bias in our velocity estimates, which alters according to glacier flow direction and topography in relation to satellite radar line of sight. This bias was not considered in [1]. Regardless of surface conditions, the effect of this bias should be zero for glaciers flowing parallel to the satellite heading angle. During a transition which encompasses wet to frozen to wet conditions, maximum apparent slow-down should be at +90 degrees (approximately aligned with typical flow of west coast glaciers) and maximum apparent speed-up at -90 degrees (approximately aligned with typical flow of east coast glaciers). When the surface changes from frozen to wet to frozen, the opposite is true: the maximum apparent speed-up should be at +90 degrees, with a maximum apparent slow-down at -90 degrees from the satellite heading angle. In each case, the apparent maximum slow-down and speed-up should be of a similar magnitude, assuming the surface returns to the pre-event state.

To estimate the impact of the phase centre depth bias on our velocity results, we compared the magnitude and sign of the speed change between consecutive image pairs to variations in the radar backscatter data. We established a winter backscatter baseline and identified the surface as ‘melted’ when the dB values were more than 4 dB below winter values [6], or ‘frozen’ if the dB values were greater than this threshold. This allowed us to compare the velocity response of our study glaciers during melt events to that which would be expected from the phase centre depth bias alone. Unfortunately, our analysis is limited by the fact that the glaciers of the Antarctic Peninsula do not flow uniformly in a radial pattern, meaning that we cannot sample equally across all flow directions. An additional complication is that surfaces may also have different wetting and refreezing patterns through time (e.g. not conforming to either a wet-frozen-wet or frozen-wet-frozen pattern).

Our analysis shows that although there are changes in ice velocity that are consistent with that expected from changes in the phase centre depth, as noted by [2], there remains a discernible signal of ice flow variations that we maintain are related to surface melting. During

many of the melt events, there is a pattern of an apparent speed-up/slow-down of ice flow perpendicular to the satellite heading angle, which we attribute to the bias effect. However, there are also substantial variations in ice flow where the flow direction is more closely aligned with the satellite heading angle, whose pattern and magnitude support the meltwater hypothesis as their cause. This is most clearly demonstrated by a new ROI from Edgeworth Glacier (Supplementary Figure 1), which flows in approximately the same direction as the satellite heading angle. Hence, any bias in the velocity data caused by melt-induced changes in phase centre depth should be close to zero for this glacier. Additional analysis of a single region of interest on this glacier reveals that short-lived (<6-day) speed-up events of approximately 200 m/year occur during periods of modelled melting (Supplementary Figure 1). These are synchronous with, and similar in characteristics to, the speed-up events reported by [1]. Surface melt features are also visible on this glacier (Supplementary Figure 1).

There are also occasions when we record a speed-up on glaciers where the surface remains wet (based on our dB analysis) across a melt event (i.e. when the surface does not refreeze between melt events that occur within consecutive velocity pairs). For example, at Jorum glacier, we observe a speed-up event during a period when the backscatter remains persistently below the winter value (Supplementary Figure 2). In these cases, we would not expect an apparent change in ice flow from the phase centre depth bias, but might expect an acceleration based on the meltwater hypothesis, given that the surface is melting throughout.

A further line of evidence suggests that the phase-centre depth bias is not greater in magnitude than the speed-up events themselves. If the penetration depth bias was the sole contributor to our observed speed-up events, we would expect any speed-up/slow-down during wetting to be balanced by an equal and opposite effect during re-freezing (as the bias effect happens in reverse). Our time-series clearly show that the majority of speed-up events produce a net positive effect as the subsequent slow-down does not offset the speed-up (e.g. Fig. 1 in main text). The exception is Cayley Glacier where there is less melt. As a result, we cannot rule out that the phase centre bias is the principal cause of velocity variations at this site.

***Supplementary Note 4* - Ice velocity errors and impact of surface melting on ice velocity**

In addition to the phase centre depth bias, [2] raise the issue of other contributing errors in the retrieval of glacier velocity that are not accounted for in [1]. The error estimate in [1] is derived from average apparent velocities over bedrock areas. The use of average velocities obtained over stable bedrock regions is frequently used to estimate measurement error for intensity tracking of radar imagery [7] or as one of a combination of methods [8]. The low-

magnitude, high-frequency variability that we observe in our velocity data is most prominent closer to the glaciers' termini, and almost absent beyond the ROI that is situated 8 km up glacier (Supplementary Figure 4 in [1]), suggesting a marine cause, possibly related to tidal sea surface height variations [1]. Unpublished ice velocity data from the western margin of the Greenland Ice Sheet, estimated using the same processing chain as used in [1], show over-winter flow variations of +/- 20 m/yr around a consistent longer-term trend suggesting that our method is capable of recording steady ice flow. That the mean speed of Flask Glacier (not studied in[1]), from GPS data mentioned in [2], and our mean speed from Jorum Glacier are similar, cannot be used as evidence that our velocity spikes are artefacts, as the transient nature of the speed-up events means they do not currently substantially affect seasonal mean velocity. It is also not clear from [2] what the units of the variability shown by the GPS-derived ice velocity data are (we are not shown the actual data). If the units of variability are 0.6 m/d (which equates to +/- 283 m/yr) the variability is not significantly different in magnitude to our observations at a similar distance up-glacier.

According to [9], there are three main sources of error for feature tracking of ice velocity using Sentinel-1 data: errors caused by inaccuracy in the cross-correlation process and mis-alignment of image pairs, errors due to ionospheric disturbances, and errors induced by geocoding (errors in the digital elevation model (DEM) used to geocode the displacements from radar to ground coordinates). Note that bias introduced by changes in the depth of the scattering phase centre is not listed as a significant source of error, nor have we been able to find any previous studies using Sentinel-1 data to quantify ice velocities that have included this in their error calculations.

The input radar data are aligned in GMTSAR to sub-pixel precision based on precise orbits and topography (https://topex.ucsd.edu/gmtsar/tar/GMTSAR_2ND_TEX.pdf). The typically low displacement values that we record over stationary regions of the radar images (as reported in [1]) suggest that the image alignment is effective. There remains the possibility that the cross-correlation procedure tracks spurious surface features that do not reflect the ice motion. Our code employs several methods to limit this, both in the pre-processing of the radar images (e.g. high pass Butterworth filter - to highlight short wave-length surface features [10]), and in the filtering of the velocity product (strain-constrained image segmentation filter, signal to noise filter, flow direction filter etc.).

Based on our processing of ice velocity data over the Greenland Ice Sheet and parts of East Antarctica, significant ionospheric disturbances create a distinct linear pattern of high displacement stripes. [9] indicate a maximum error related to such features of 0.25 m/d (~91

m/yr). We do not, however, see evidence of significant ionospheric effects in our Antarctic Peninsula data, yet we nevertheless include in our code a filter to minimise such artefacts if detected (https://www.math.univ-toulouse.fr/~weiss/).

We used the ASTER GDEM 100-m Digital Elevation Model of the Antarctic Peninsula to geocode the results of our cross-correlations. The DEM has a mean elevation difference of -4 m (±25 m Root Mean Square Error) from co-located ICESat data. The phase centre depth bias is equivalent to a change in the DEM of up to approximately +/- 5 m across the time period of our image baselines. Supplementary Figure 1 from [2] suggests a maximum related error of 0.5 - 1 m/d for ice flow perpendicular to the satellite heading angle, assuming that the entirety of their observed change in ice speed is due to the bias. The magnitude of this error is expected to reduce to zero for ice flow parallel to the satellite heading angle, but will also vary based on several additional factors: the spatial distribution of firn thickness; surface topography; and the effect of variations in the degree of surface melting between radar data acquisitions. As such it is difficult to quantify satisfactorily.

Overall, the largest reliably quantifiable potential error comes from ionospheric disturbances, which according to [9] has a maximum value of 91 m/yr. We have implemented this error figure in our new analysis (Fig. 1 in main text; Supplementary Figures 1 and 2). Our speed-up events are typically much greater in magnitude than this.

Supplementary Tables:

Supplementary Table 1: Observations of sea ice from Sentinel 1 imagery during two speed up events reported in Tuckett et al. (2019).

**Location**	**March 2017 speed-up**			**November 2017 speed-up**			**March 2018 speed-up**		
	Prior (24/03)	During (26/03)	After (01/04)	Prior (28/10)	During (03/11)	After (07/11)	Prior (21/03)	During (27/03)	After (31/03)
Cayley glacier front	No Sea ice neighbouring to glacier	Some melange in bay	Melange extent reduced in bay	Sea ice away from glacier front	Sea ice away from glacier front	No sea ice in vicinity	Sea ice neighbouring to glacier front	Area of sea ice increases	Area of sea ice increases further
Drygalski glacier front	Polyna developed at front	Melange neighbouring to glacier	Open water polyna.	Sea ice at glacier front	Sea ice at glacier front	Sea ice at glacier front	Sea ice neighbouring glacier front	Polyna neighbours glacier	Sea ice partially fills polyna, but discontinuous and open water next to glacier.
Hektoria glacier front	Sea ice neighbouring to glacier front	Sea ice unchanged	Sea ice unchanged	Sea ice at glacier front	Sea ice unchanged	Sea ice unchanged	Sea ice at glacier front	Sea ice unchanged	Sea ice unchanged
Jorum glacier front	Sea ice neighbouring to glacier front	Sea ice unchanged	Sea ice unchanged	Sea ice at glacier front	Sea ice unchanged	Sea ice unchanged	Sea ice at glacier front	Sea ice unchanged	Sea ice unchanged
Crane glacier front	Sea ice neighbouring to glacier front	Sea ice unchanged	Sea ice unchanged	Sea ice at glacier front	Sea ice unchanged	Sea ice unchanged	Sea ice at glacier front	Sea ice unchanged	Sea ice unchanged
Larsen B embayment	Open water at edge of embayment. Large sea ice area pinned in bay.	Little to no change in pinned ice extent. Movement of sea ice unattached to pinned ice.	Further opening of open ocean at edge of embayment.	Large area pinned. Some cracks in sea ice developing far from glaciers.	Cracks healed. Sea ice extent increased.	Open ocean forming next to pinned ice.	Large area of sea ice pinned in bay. Thinner sea ice neighbouring further from glacier fronts	Movement of sea ice as reported in Rott et al. (submitted).	Further movement of sea ice away from embayment.

Supplementary Figures:

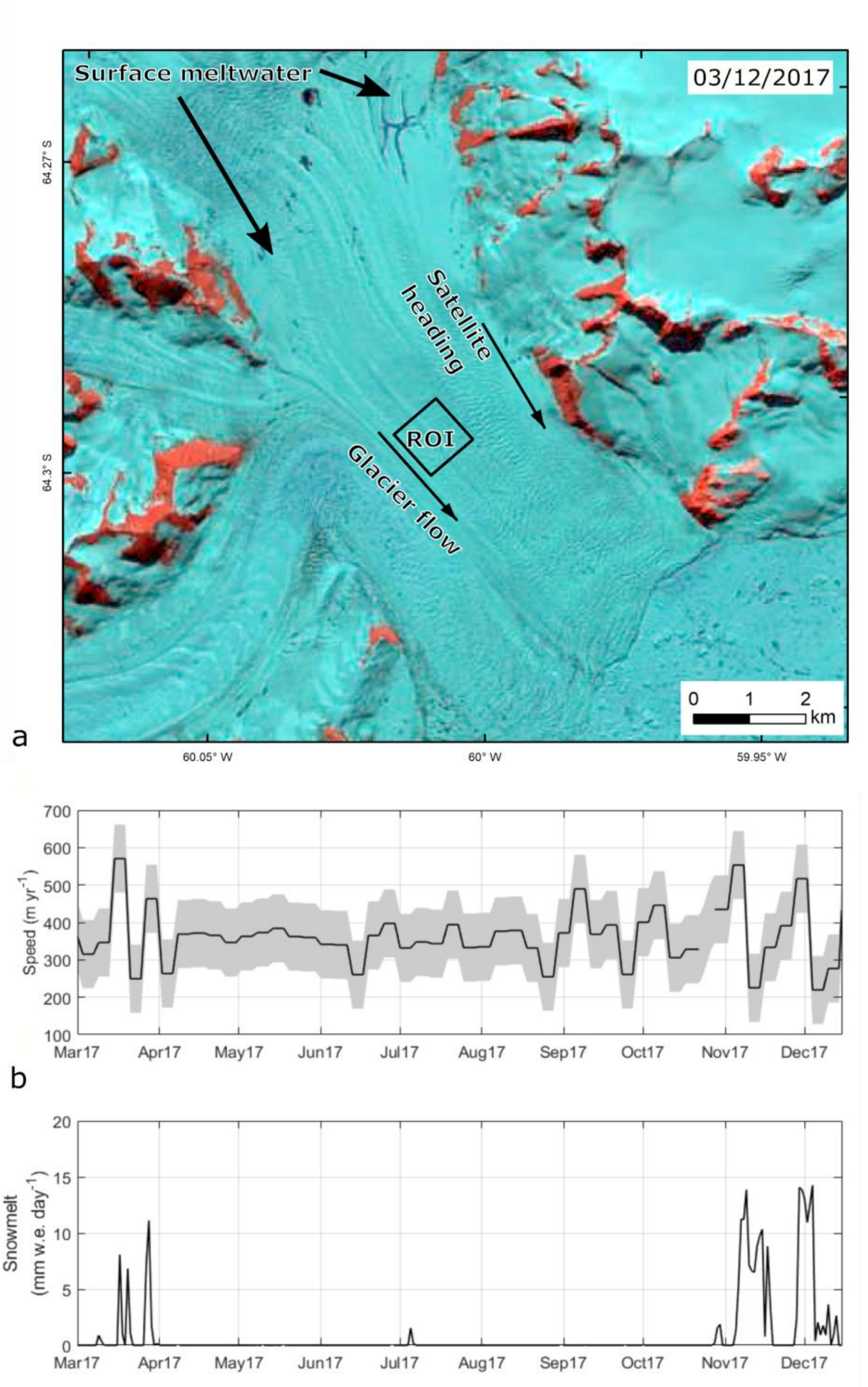

Supplementary Figure 1. Location of Edgeworth glacier and indication of speed-up events. a) Landsat image of Edgeworth glacier. Note the presence of meltwater features high-up on the glacier. The region of interest (ROI) studied here contains ice flowing nearly parallel to the satellite heading. b) Surface speed and modelled melt from the ROI. Note the presence of speed-up events during melt events.

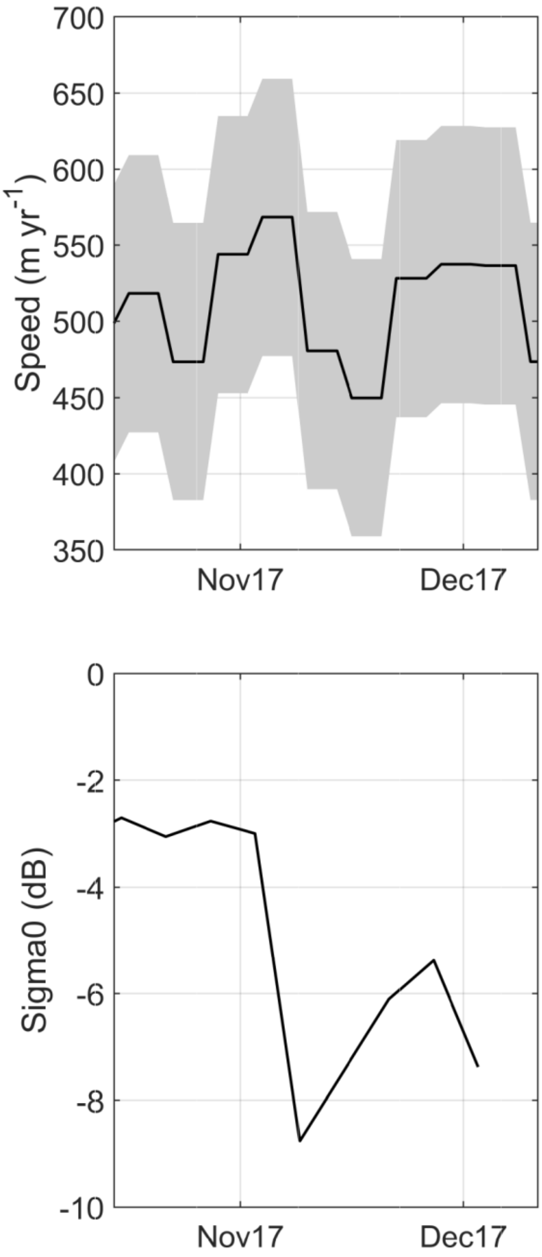

Supplementary Figure 2. Changes in backscatter for speed-up events at Jorum glacier. A speed-up event occurs during persistent melting conditions (i.e. 4 dB below the winter mean).

Supplementary References:

1. Tuckett, P.A., Ely, J.C., Sole, A.J., Livingstone, S.J., Davison, B.J., van Wessem, J.M. and Howard, J., 2019. Rapid accelerations of Antarctic Peninsula outlet glaciers driven by surface melt. *Nature Communications*, *10*(1), pp.1-8.
2. Rott, H., Wuite, J., De Rydt, J., Gudmundsson, G.H., Floricioiu, D. and Rack, W. Impact of marine processes on flow dynamics of northern Antarctic Peninsula outlet glaciers. *Nature Communications* (accepted).
3. Dierking, W., 2009. Mapping of different sea ice regimes using images from Sentinel-1 and ALOS synthetic aperture radar. Ieee transactions on geoscience and remote sensing, 48(3), pp.1045-1058.
4. Howat, I. M., Porter, C., Smith, B. E., Noh, M.-J., and Morin, P.: The Reference Elevation Model of Antarctica, The Cryosphere, 13, 665-674, https://doi.org/10.5194/tc-13-665-2019, 2019.
5. Friedl, P., Weiser, F., Fluhrer, A. and Braun, M.H., 2019. Remote sensing of glacier and ice sheet grounding lines: A review. Earth-Science Reviews, p.102948.
6. Zhou, C. and Zheng, L., 2017. Mapping radar glacier zones and dry snow line in the Antarctic peninsula using sentinel-1 images. Remote Sensing, 9(11), p.1171.
7. Sundal, A.V., Shepherd, A., Nienow, P., Hanna, E., Palmer, S. and Huybrechts, P., 2011. Melt-induced speed-up of Greenland ice sheet offset by efficient subglacial drainage. Nature, 469(7331), pp.521-524.
8. Rott, H., Rack, W., Skvarca, P. and De Angelis, H., 2002. Northern Larsen ice shelf, Antarctica: Further retreat after collapse. *Annals of Glaciology*, *34*, pp.277-282.
9. Nagler, T., Rott, H., Hetzenecker, M., Wuite, J. and Potin, P., 2015. The Sentinel-1 mission: New opportunities for ice sheet observations. Remote Sensing, 7(7), pp.9371-9389.
10. de Lange, R., Luckman, A. and Murray, T., 2007. Improvement of satellite radar feature tracking for ice velocity derivation by spatial frequency filtering. IEEE Transactions on Geoscience and Remote Sensing, 45(7), pp.2309-2318.
